# Diastolic Dysfunction Is an Independent Predictor of Cardiovascular Events in Incident Dialysis Patients with Preserved Systolic Function

**DOI:** 10.1371/journal.pone.0118694

**Published:** 2015-03-04

**Authors:** Jae Hyun Han, Ji Suk Han, Eun Jin Kim, Fa Mee Doh, Hyang Mo Koo, Chan Ho Kim, Mi Jung Lee, Hyung Jung Oh, Jung Tak Park, Seung Hyeok Han, Dong-Ryeol Ryu, Tae-Hyun Yoo, Shin-Wook Kang

**Affiliations:** 1 Department of Internal Medicine, Yonsei University College of Medicine, Seoul, Korea; 2 Department of Internal Medicine, School of Medicine, Ewha Womans University, Seoul, Korea; 3 Severance Biomedical Science Institute, Brain Korea 21 PLUS project for Medical Science, Yonsei University College of Medicine, Seoul, Korea; Children’s Hospital Boston/Harvard Medical School, UNITED STATES

## Abstract

**Background:**

Diastolic heart failure (HF), the prevalence of which is gradually increasing, is associated with cardiovascular (CV) morbidity and mortality in the general population and, more specifically, in patients with end-stage renal disease (ESRD). However, the impact of diastolic dysfunction on CV outcomes has not been studied in incident dialysis patients with preserved systolic function.

**Methods:**

This prospective observational cohort study investigates the clinical consequence of diastolic dysfunction and the predictive power of diastolic echocardiographic parameters for CV events in 194 incident ESRD patients with normal or near normal systolic function, who started dialysis between July 2008 and August 2012.

**Results:**

During a mean follow-up duration of 27.2 months, 57 patients (29.4%) experienced CV events. Compared to the CV event-free group, patients with CV events had a significantly higher left ventricular (LV) mass index, ratio of early mitral flow velocity (E) to early mitral annulus velocity (E’) (E/E’), LA volume index (LAVI), deceleration time, and right ventricular systolic pressure, and a significantly lower LV ejection fraction and E’. In multivariate Cox proportional hazard analysis, E/E’>15 and LAVI>32 mL/m^2^ significantly predicted CV events (E/E’>15: hazard ratio [HR] = 5.40, 95% confidence interval [CI] = 2.73–10.70, *P*< .001; LAVI>32 mL/m^2^: HR = 5.56, 95% CI = 2.28–13.59, *P*< .001]. Kaplan-Meier analysis revealed that patients with both E/E’>15 and LAVI>32mL/m^2^ had the worst CV outcomes.

**Conclusion:**

An increase in E/E’ or LAVI is a significant risk factor for CV events in incident dialysis patients with preserved LV systolic function.

## Introduction

Diastolic heart failure (HF) is defined by the signs and symptoms of heart failure, along with left ventricular (LV) diastolic dysfunction and normal or mildly impaired LV systolic function.[1,2] A number of previous studies have shown that diastolic HF accounts for one-third to half of the cases of HF in the general population, and its prevalence is steadily increasing.[3–5] In addition, Redfield et al. have shown that diastolic HF was even more common than HF with reduced ejection fraction (EF).[5] Patients with diastolic HF were also found to have significantly higher morbidity and mortality compared to the general population.[5–7] Moreover, the clinical outcomes of patients with diastolic HF were shown to be comparable with those of HF patients with reduced systolic function.[4,8]

Several conditions that are prevalent in patients with end-stage renal disease (ESRD)—such as hypertension, diabetes mellitus (DM), coronary artery disease, and anemia—are known to be implicated in the pathogenesis of diastolic dysfunction. In ESRD patients, chronic volume overload, oxidative stress, inflammation, and abnormal mineral metabolism have been shown to contribute to the development of diastolic dysfunction.[9–11] Thus, diastolic dysfunction and left ventricular hypertrophy (LVH) are the most common echocardiographic findings in patients with ESRD.[9]

Diastolic dysfunction can be diagnosed by Doppler echocardiography. Echocardiography is a commonly used to confirm cardiovascular risk and to guide treatment in ESRD patients on dialysis.[12] Earlier studies on LV mass index (LVMI), LVEF, and LV chamber volume have provided valuable information on these patients. Recent studies have shown that diastolic dysfunction, defined as the increased ratio of early mitral flow velocity (E) to early mitral annulus velocity (E’) (E/E’) and/or high left atrium (LA) volume index (LAVI), is also an independent predictor of mortality in chronic dialysis patients.[13,14] However, the impact of diastolic dysfunction on clinical outcomes has rarely been investigated in new ESRD patients starting dialysis. Furthermore, the potential association in this population between diastolic dysfunction with preserved LV systolic function and poor cardiovascular outcomes has not been previously investigated.

Therefore, in the present study, we aimed to determine the clinical consequences of diastolic dysfunction and the echocardiographic parameters of diastolic dysfunction that can be used to significantly predict cardiovascular events in incident dialysis patients with preserved LV systolic function.

## Methods

### Patients

For this prospective observational cohort study, we initially recruited 293 patients who started hemodialysis (HD) or peritoneal dialysis (PD) at the Yonsei University Health System, Seoul, Korea, between July 2008 and August 2012. We excluded 24 patients who did not undergo echocardiography due to noncompliance or other personal reasons. Of the remaining 269 patients, 75 patients were excluded for the following reasons: previous history of cardiac surgery (n = 6) or kidney transplantation (n = 16), moderate to severe valvular heart disease (n = 9), active infection (n = 8), active malignancy (n = 5), and follow-up duration of less than 3 months (n = 6). In addition, 25 patients with LV systolic dysfunction (EF<50%) were also excluded. Thus, a total of 194 patients were included in the final analysis (S1 Fig.).

The study was performed in accordance with the Declaration of Helsinki and approved by the Institutional Review Board of Yonsei University Health System Clinical Trial Center. Informed written consent was obtained from each patient before participation in the study.

### Data collection

Demographic and clinical data including age, gender, comorbidities, and medications, were recorded at dialysis initiation. Cardiovascular (CV) disease was defined as a history of coronary, arrhythmia, peripheral vascular disease, or cerebrovascular; coronary arterial disease (CAD) was defined as a history of percutaneous coronary intervention, coronary artery bypass grafts, myocardial infarction, or angina; cerebrovascular disease was defined as a history of transient ischemic attack, ischemic stroke, cerebral hemorrhage, or carotid endarterectomy; and peripheral vascular disease was defined as a history of claudication, ischemic limb loss and/or gangrene, or peripheral revascularization procedure. Cardiovascular events were designated as events requiring hospitalization or going to the emergency room because of cardiovascular disease. Laboratory data were measured using fasting blood samples obtained close to the time of discharge when the patients were considered to be clinically stable and in an euvolemic state, and were drawn prior to the start of a midweek HD session in HD patients and 2 hours after the first PD exchange with 1.5% dextrose dialysate in PD patients. The following data were measured: levels of hemoglobin, blood urea nitrogen, serum creatinine, calcium, phosphorus, albumin, total cholesterol, triglyceride, intact parathyroid hormone (iPTH), alkaline phosphatase, sodium, potassium, bicarbonate, iron, ferritin, high-sensitivity C-reactive protein (hs-CRP), N-terminal proB-type natriuretic peptide (NT-proBNP), cardiac troponin T (cTnT). Moreover, 24-hour urine collection was performed to determine the residual urine volume, 24-hour urinary protein, urea, and creatinine excretion values. Ultrafiltration was defined as the amount of fluid removed during the mid-week HD session to achieve dry weight in HD patients and as the net drained volume of PD fluid for 24 hours in PD patients. Ultrafiltration was determined every 3 months in both HD and PD patients.

### Echocardiography

Echocardiographic examinations were performed close to the time of discharge on a non-dialysis day for HD patients and on the day before the discharge date with an empty abdomen for PD patients, based on the imaging protocol recommended by the American Society of Echocardiography.[15] Comprehensive echocardiographic measurements were obtained using an ultrasound machine (Vivid 7; GE Vingmed Ultrasound AS, Horten, Norway) with a 2.5 MHz probe. LVEF, an indicator of LV systolic function, was calculated using a modified biplane Simpson’s method from the apical two-chamber and four-chamber views. LV mass was determined based on the area-length process using the method described by Devereux et al.[16] and LVMI was calculated by dividing the LV mass by the body surface area. Mitral inflow was measured by Doppler echocardiography from the apical four-chamber view, with the Doppler beam aligned parallel to the direction of flow and with a 1–2 mm sample volume placed between the tips of the mitral leaflets during diastole. The mitral inflow profiles were used to measure the peak mitral inflow velocities at the early (E) and late (A) diastole, its deceleration time (DT), and the isovolumetric relaxation time. Doppler tissue imaging of the mitral annulus was also obtained. From the apical four-chamber view, a 1–2 mm sample volume was placed at the septal and lateral mitral annulus, and the average of the two values was used to evaluate the early (E’) and late (A’) diastolic peak velocities. Moderate to severe diastolic dysfunction was defined as E/E’>15.[2] LA volume was assessed using the biplane area-length method from the apical two-chamber and four-chamber views and was indexed for body surface area. Measurements were obtained in end-systole from the frame preceding mitral valve opening. A moderately to severely enlarged LA was defined as LAVI>32 mL/m^2^.[17,18] Right ventricular systolic pressure (RVSP) was calculated using the modified Bernoulli equation [4 × (tricuspid systolic jet)^2^ + right atrial pressure (RAP) mmHg]. RAP was estimated by the guidelines of the American Society of Echocardiography.[19]

### Statistical analysis

Statistical analyses were performed using SPSS for Windows, version 18.0 (SPSS, Inc., Chicago, IL, USA). Continuous variables were expressed as mean ± standard deviation or median (interquartile range), and categorical variables as a number (percentage). Patients were divided into two groups according to the presence of CV events—the CV event-free group and CV event group. The baseline characteristics were compared between these two groups using Student’s *t*-test or Mann-Whitney U tests for continuous variables and the chi-square test for categorical variables. Furthermore, the relationships between echocardiographic parameters were determined by Pearson’s correlation analysis. Cumulative survival curves were generated by the Kaplan-Meier method, and between-group survival was compared by a log-rank test. The independent prognostic values of LAVI and E/E’ for CV events were ascertained by multivariate Cox proportional hazards regression analysis, which included variables with a *P* value of <. 10 in the univariate Cox analysis as well as traditional CV risk factors. In addition, the predictive values of E/E’ and LAVI for CV events were compared using the calculated area under the receiver operating characteristic curve (AUC). *P* values of <. 05 were considered statistically significant.

## Results

### Patient characteristics

The baseline patient characteristics are shown in Table 1. The mean age was 57.9 ± 14.5 years, and 114 patients (58.8%) were male. The most common cause of ESRD was DM (46.9%), followed by hypertension (22.7%). A total of 155 patients (79.9%) were treated with HD and 39 patients (20.1%) were treated with PD. All PD patients started with continuous ambulatory peritoneal dialysis patients. During the follow-up duration, however, 5 patients out of 39 patients changed to automated PD. When the patients were divided into two groups according to dialysis modality, HD patients were significantly older than PD patients (*P* = .001) and significantly more patients were taking aspirin in the HD group compared to patients on PD (*P* = .001), while serum creatinine concentrations were significantly higher in PD patients compared to the HD group (*P* = .009). However, there were no significant differences in the other baseline characteristics between the two groups.

**Table 1:** Baseline characteristics of the study patients.

Variables	Total (n = 194)	HD (n = 155)	PD (n = 39)	*P*
Age (years)	57.89 ± 14.53	59.55 ± 14.36	51.30 ± 13.44	0.001
Sex (male)	114 (58.8)	93 (60.0)	21 (53.8)	0.302
BMI (kg/m2)	23.60 ± 3.41	23.6 ± 3.37	23.46 ± 3.64	0.787
Smoking (Yes)	29 (14.9)	22 (14.2)	7 (17.9)	0.357
Systolic BP (mmHg)	141.61 ± 18.69	142.38 ± 18.39	138.59 ± 19.80	0.259
Diastolic BP (mmHg)	77.60 ± 13.00	77.54 ± 12.08	77.85 ± 16.34	0.896
Primary cause of renal disease				0.381
Diabetes	91 (46.9)	72 (46.5)	19 (48.7)	
Hypertension	44 (22.7)	35 (22.6)	9 (20.5)	
GN	23 (11.9)	19 (12.3)	4 (10.3)	
Others	10 (5.2)	7 (4.5)	3 (7.7)	
unknown	26 (13.4)	22 (14.2)	4 (10.3)	
Comorbid disease				
Chronic lung disease	20 (10.4)	18 (11.7)	2 (5.1)	0.264
Coronary arterial disease	27 (13.9)	25 (16.1)	2 (5.1)	0.117
Arrhythmia	10 (5.2)	9 (5.8)	1 (2.6)	0.690
Peripheral arterial disease	33 (17.0)	25 (16.1)	8 (20.5)	0.633
Cerebrovascular disease	35 (18.0)	32 (20.6)	3 (7.7)	0.065
Ulcer	11 (5.7)	6 (3.9)	5 (12.8)	0.046
Liver disease	5 (2.6)	4 (2.6)	1 (2.6)	0.995
DM	111 (57.2)	88 (56.8)	23 (59.0)	0.858
Modified CCI	5.23 ± 2.62	5.42 ± 2.65	4.47 ± 2.43	0.052
Davies score	1.07 ± 0.95	1.13 ± 0.99	0.83 ± 0.74	0.092
Baseline laboratory findings				
Hemoglobin (g/dL)	9.16 ± 5.88	9.26 ± 6.55	8.73 ± 1.25	0.614
Blood urea nitrogen (mg/dL)	57.55 ± 28.54	56.69 ± 29.87	60.99 ± 22.49	0.401
Serum creatinine (mg/dL)	8.09 ± 10.01	7.16 ± 2.88	11.84 ± 21.38	0.009
Serum albumin (g/dL)	3.31 ± 1.44	3.34 ± 1.58	3.29 ± 0.62	0.661
Total cholesterol (mg/dL)	149.97 ± 40.68	148.64 ± 39.38	155.28 ± 45.64	0.364
Triglyceride (mg/dL)	113.15 ± 48.68	110.97 ± 44.36	121.84 ± 62.66	0.213
LDL-C (mg/dL)	81.69 ± 29.49	79.62 ± 28.68	89.93 ± 31.54	0.051
HDL-C (mg/dL)	37.94 ± 12.25	37.96 ± 12.59	37.87 ± 10.94	0.965
Sodium (mmol/L)	137.62 ± 3.81	137.79 ± 3.57	136.97 ± 4.67	0.232
Potassium (mmol/L)	4.10 ± 0.76	4.13 ± 0.80	3.97 ± 0.59	0.263
Bicarbonate (mmol/L)	22.84 ± 4.36	22.80 ± 4.10	22.99 ± 5.30	0.812
iPTH (pmol/L)	195.01 (99.85–231.5)	191.92 (98.0–230.96)	207.24 (118.0–234.0)	0.630
hs-CRP (mg/L)	16.03 (1–18.72)	16.99 (1.02–19.03)	12.14 (1.01–14.05)	0.353
NT-proBNP (pg/mL)	12213 (1924–20682)	12549 (2101–21047)	10033 (605–19439)	0.434
cTnT (ng/mL)	0.10 (0.02–0.10)	0.11 (0.03–0.12)	0.05 (0.02–0.07)	0.076
24-hr urine related parameter				
Urine volume (mL/day)	886.0 (467.5–1172.5)	868.9 (460.0–1150.0)	953.8 (470.0–1400.0)	0.412
Urine protein (mg/day)	2103.6 (556.3–2721.5)	2224.5 (532.1–2863.4)	1623.3 (670.7–2184.0)	0.132
Medications				
RAS blocker	155 (79.9)	123 (79.4)	32 (82.1)	0.825
Diuretics	118 (60.8)	95 (61.3)	23 (59.0)	0.855
CCB	132 (68)	108 (69.7)	24 (61.5)	0.342
Beta blocker	120 (61.9)	96 (61.9)	24 (61.5)	0.998
Aspirin	51 (26.3)	49 (31.6)	2 (5.1)	0.001
Clopidogrel	17 (8.8)	16 (10.3)	1 (2.6)	0.363
Vitamin D	97 (50.0)	71 (45.8)	26 (66.7)	0.031
ESA	167 (86.1)	133 (85.8)	4 (87.2)	1.000

*HD*, hemodialysis; *PD*, peritoneal dialysis; *BMI*, body mass index; *BP*, blood pressure; *GN*, glomerulonephritis; *DM*, diabetes mellitus; *CCI*, Charlson comorbidity index; *HDL-C*, high-density lipoprotein cholesterol; *LDL-C*, low-density lipoprotein cholesterol; *iPTH*, intact parathyroid hormone; *hs-CRP*, high-sensitivity C reactive protein; *NT-proBNP*, N-terminal pro B-type natriuretic peptide; *cTnT*, cardiac troponin T; *RAS*, renin-angiotensin-system; *CCB*, calcium channel blocker; *ESA*, erythropoietin stimulating agent.

Data are expressed as mean ± SD, number (percentage), or median (range).

### Clinical and laboratory findings according to the presence of CV events

During a mean follow-up duration of 27.2 months, 57 patients (29.4%) experienced CV events. We divided the patients into two groups according to the presence of CV events, and compared the baseline clinical and laboratory findings between the two groups (Table 2). The following variables were significantly higher in the CV event group than in the CV event-free group: age; the proportion of patients with DM, CAD, arrhythmia, cerebrovascular disease, or chronic lung disease; systolic blood pressure (BP); 24-hour urine volume; serum NT-proBNP concentrations, and the proportion of patients receiving clopidogrel. In contrast, there were no significant differences in gender; dialysis modality; the proportion of patients with peripheral artery disease; diastolic BP; and levels of hemoglobin, calcium, phosphate, albumin, iPTH, hs-CRP, and cTnT between the two groups. The use of anti-hypertensive agents, vitamin D medication, and erythropoiesis-stimulating agents were also comparable between the two groups.

**Table 2:** Baseline characteristics of the study patients according to the presence of CV events.

Variables	Total (n = 194)	CV event (-) (n = 137)	CV event (+) (n = 57)	*P*
Age (years)	57.89 ± 14.53	56.25 ± 14.91	61.84 ± 12.85	0.014
Sex (male)	114 (58.8)	81 (59.1)	33 (57.9)	0.874
Dialysis modality (HD)	155 (79.9)	105 (76.6)	50 (87.7)	0.114
BMI (kg/m2)	23.60 ± 3.41	23.32 ± 3.55	24.27 ± 3.00	0.081
Smoking (Yes)	29 (14.9)	20 (14.6)	9 (15.8)	0.832
Systolic BP (mmHg)	141.61 ± 18.69	139.36 ± 18.84	147.05 ± 17.32	0.009
Diastolic BP (mmHg)	77.60 ± 13.00	77.22 ± 13.16	78.52 ± 16.68	0.525
Primary cause of renal disease				0.103
Diabetes	91 (46.9)	56 (40.9)	35 (61.4)	
Hypertension	44 (22.7)	34 (24.8)	10 (17.5)	
GN	23 (11.9)	18 (13.1)	5 (8.8)	
Others	10 (5.2)	7 (5.1)	3 (5.3)	
unknown	26 (13.4)	22 (16.1)	4 (7.0)	
Comorbid disease				
Chronic lung disease	20 (10.4)	10 (7.4)	10 (17.5)	0.041
Coronary arterial disease	27 (13.9)	13 (9.5)	14 (24.6)	0.008
Arrhythmia	10 (5.2)	3 (2.2)	7 (12.3)	0.008
Peripheral arterial disease	33 (17.0)	26 (19.0)	7 (12.3)	0.3
Cerebrovascular disease	35 (18.0)	16 (11.7)	19 (33.3)	0.001
Ulcer	11 (5.7)	8 (5.8)	3 (5.3)	0.874
Liver disease	5 (2.6)	4 (2.9)	1 (1.8)	0.641
DM	111 (57.2)	71 (51.8)	40 (70.2)	0.025
Modified CCI	5.23 ± 2.62	5.05 ± 2.68	5.68 ± 2.47	0.133
Davies score	1.07 ± 0.95	0.98 ± 0.90	1.28 ± 1.03	0.055
Baseline laboratory findings				
Hemoglobin (g/dL)	9.16 ± 5.88	8.69 ± 1.16	10.30 ± 10.70	0.083
Blood urea nitrogen (mg/dL)	57.55 ± 28.54	59.17 ± 29.57	53.67 ± 25.74	0.223
Serum creatinine (mg/dL)	8.09 ± 10.01	8.80 ± 11.76	6.41 ± 2.31	0.131
Serum albumin (g/dL)	3.31 ± 1.44	3.43 ± 1.67	3.06 ± 0.48	0.102
Total cholesterol (mg/dL)	149.97 ± 40.68	148.19 ± 38.08	154.28 ± 46.42	0.343
Triglyceride (mg/dL)	113.15 ± 48.68	110.32 ± 49.20	119.98 ± 46.85	0.208
LDL-C (mg/dL)	81.69 ± 29.49	81.27 ± 27.39	82.72 ± 34.25	0.757
HDL-C (mg/dL)	37.94 ± 12.25	38.60 ± 12.25	36.39 ± 12.21	0.253
Sodium (mmol/L)	137.62 ± 3.81	138.00 ± 3.68	136.72 ± 4.00	0.032
Potassium (mmol/L)	4.10 ± 0.76	4.09 ± 0.76	4.11 ± 0.79	0.851
Bicarbonate (mmol/L)	22.84 ± 4.36	22.89 ± 4.43	22.70 ± 4.20	0.779
iPTH (pmol/L)	195.01 (99.85–231.5)	209.37 (100.0–235.05)	159.90 (91.44–213.96)	0.077
hs-CRP (mg/L)	16.03 (1–18.72)	14.24 (1–13.94)	20.30 (1.85–25.25)	0.182
NT-proBNP (pg/mL)	12213 (1924–20682)	9356 (1338–16737.5)	17228 (3512–35000)	<0.001
cTnT (ng/mL)	0.10 (0.02–0.10)	0.083 (0.024–0.099)	0.144 (0.03–0.126)	0.054
24-hr urine related parameter				
Urine volume (mL/day)	886.0 (467.5–1172.5)	978.0 (560.0–1360.0)	665.1 (375.0–890.0)	<0.001
Urine protein (mg/day)	2103.6 (556.3–2721.5)	2048.6 (554.2–2617.1)	2236.1 (559.8–2793.7)	0.595
Medications				
RAS blocker	155 (79.9)	108 (78.8)	47 (82.5)	0.695
Diuretics	118 (60.8)	83 (60.6)	35 (61.4)	0.915
CCB	132 (68)	94 (68.6)	38 (66.7)	0.866
Beta blocker	120 (61.9)	81 (59.1)	39 (68.4)	0.258
Aspirin	51 (26.3)	33 (24.1)	18 (31.6)	0.288
Clopidogrel	17 (8.8)	8 (5.8)	9 (15.8)	0.016
Vitamin D	97 (50)	70 (51.1)	27 (47.4)	0.753
ESA	167 (86.1)	118 (86.1)	49 (86.0)	0.976

*CV*, cardiovascular; *HD*, hemodialysis; *BMI*, body mass index; *BP*, blood pressure; *GN*, glomerulonephritis; *DM*, diabetes mellitus; *CCI*, Charlson comorbidity index; *HDL-C*, high-density lipoprotein cholesterol; *LDL-C*, low-density lipoprotein cholesterol; *iPTH*, intact parathyroid hormone; *hs-CRP*, high-sensitivity C reactive protein; *NT-proBNP*, N-terminal pro B-type natriuretic peptide; *cTnT*, cardiac troponin T; *RAS*, renin-angiotensin-system; *CCB*, calcium channel blocker; *ESA*, erythropoietin stimulating agent.

Data are expressed as mean ± SD, number (percentage), or median (range).

### Echocardiographic parameters according to the presence of CV events

Table 3 presents the echocardiographic parameters of the two groups. Compared to the CV event-free group, LV end-diastolic diameter (LVEDD), LVMI, E/E’, DT, LA volume and LAVI, and RVSP were significantly higher, whereas LVEF and E’ were significantly lower in patients with CV events. However, there were no significant differences in LV posterior wall thickness, interventricular septal thickness, LV mass, E/A, and right atrial pressure between the two groups.

**Table 3:** Baseline echocardiographic parameters of the study patients according to the presence of CV events.

Variables	Total (n = 194)	CV event (-) (n = 137)	CV event (+) (n = 57)	*P*
LVEF (%)	64.42 ± 7.69	65.61 ± 7.23	61.57 ± 8.08	0.001
LVEDD (mm)	52.71 ± 6.25	51.77 ± 5.69	54.98 ± 6.95	0.001
LVESD (mm)	35.91 ± 6.12	34.92 ± 5.61	38.28 ± 6.67	0.003
IVST (mm)	11.57 ± 6.55	11.71 ± 7.74	11.22 ± 1.51	0.638
PWT (mm)	11.00 ± 1.52	11.00 ± 1.55	11.00 ± 1.49	0.976
LVM (g)	241.72 ± 145.47	238.87 ± 166.75	248.68 ± 71.06	0.672
LVMI (g/m^2^)	138.47 ± 34.29	135.09 ± 33.12	146.73 ± 35.96	0.032
E/E’	15.73 ± 7.04	13.74 ± 6.19	20.51 ± 6.69	<0.001
E’ (cm/sec)	5.45 ± 1.95	5.67 ± 2.09	4.92 ± 1.47	0.016
E/A	1.02 ± 0.56	1.00 ± 0.55	1.04 ± 0.56	0.730
DT (msec)	208.44 ± 62.99	214.15 ± 65.81	194.46 ± 53.45	0.048
LAD (mm)	42.05 ± 7.62	40.19 ± 6.70	46.52 ± 7.88	<0.001
LAV (mL)	68.20 ± 31.97	61.04 ± 23.30	85.41 ± 42.15	<0.001
LAVI (mL/m^2^)	40.22 ± 18.35	35.87 ± 13.93	50.68 ± 23.05	<0.001
RVSP (mmHg)	30.40 ± 11.81	28.35 ± 9.38	35.35 ± 15.21	<0.001
RAP (mmHg)	6.13 ± 3.04	5.91 ± 3.09	6.66 ± 2.88	0.116

*CV*, cardiovascular; *LVEF*, left ventricular ejection fraction; *LVEDD*, left ventricular end diastolic diameter; *LVESD*, left ventricular end systolic diameter; *IVST*, interventricular septal thickness; *PWT*, posterior wall thickness; *LVMI*, left ventricular mass index; *LAD*, left atrial diameter; *LAVI*, left atrial volume index; *DT*, deceleration time; *RVSP*, right ventricular systolic pressure; *RAP*, right atrial pressure

Data are presented as mean ± SD.

### Correlations between echocardiographic parameters

Pearson’s correlation analyses revealed that there was a significant positive correlation between E/E’ and LAVI. In addition, E/E’ and LAVI had significant positive correlations with LAD, E/A, LVMI, RVSP, and RAP, and a significant inverse relationship with DT (Table 4).

**Table 4:** Correlations of E/E’ and LAVI with other echocardiographic parameters.

		LVEF	LVEDD	LVESD	LVMI	E/E’	LAVI	E’	E/A	DT	LAD	RVSP	RAP
LVEF	γ	1	–0.197	–0.076	–0.068	–0.167	–0.136	0.101	–0.070	0.214	–0.165	–0.071	–0.096
	P		0.006	0.292	0.345	0.020	0.058	0.162	0.342	0.003	0.022	0.325	0.185
LVEDD	γ		1	0.054	0.068	0.111	0.069	– 0.065	–0.012	–0.111	0.062	–0.013	0.002
	P			0.450	0.347	0.125	0.337	0.369	0.869	0.125	0.387	0.862	0.978
LVESD	γ			1	0.087	0.014	0.134	0.070	0.056	0.035	0.120	0.110	0.034
	P				0.227	0.849	0.062	0.335	0.447	0.631	0.096	0.127	0.635
LVMI	γ				1	0.290	0.498	–0.224	0.109	0.069	0.437	0.203	0.069
	P					<0.001	<0.001	0.002	0.139	0.340	<0.001	0.005	0.342
E/E’	γ					1	0.462	–0.494	0.217	–0.152	0.429	0.381	0.166
	P						<0.001	<0.001	0.003	0.035	<0.001	<0.001	0.021
LAVI	γ						1	–0.129	0.400	–0.152	0.840	0.526	0.251
	P							0.073	<0.001	0.035	<0.001	<0.001	<0.001
E	γ							1	0.206	–0.232	–0.209	–0.029	–0.016
	P								0.005	0.001	0.004	0.690	0.827
E/A	γ								1	–0.366	0.340	0.353	0.189
	P									<0.001	<0.001	<0.001	0.010
DT	γ									1	0.151	–0.261	–0.112
	P										0.036	<0.001	0.122
LAD	γ										1	0.444	0.222
	P											<0.001	0.002
RVSP	γ											1	0.350
	P												<0.001
RAP	γ												1
	P												

*LVEF*, left ventricular ejection fraction; *LAVI*, left atrial volume index; *LAD*, left atrial diameter; *DT*, deceleration time; *LVMI*, left ventricular mass index; *LVESD*, left ventricular end systolic diameter; *LVEDD*, left ventricular end diastolic diameter; *RVSP*, right ventricular systolic pressure; *RAP*, right atrial pressure

### CV events according to clinical, laboratory, and echocardiographic findings

**Fig 1:**
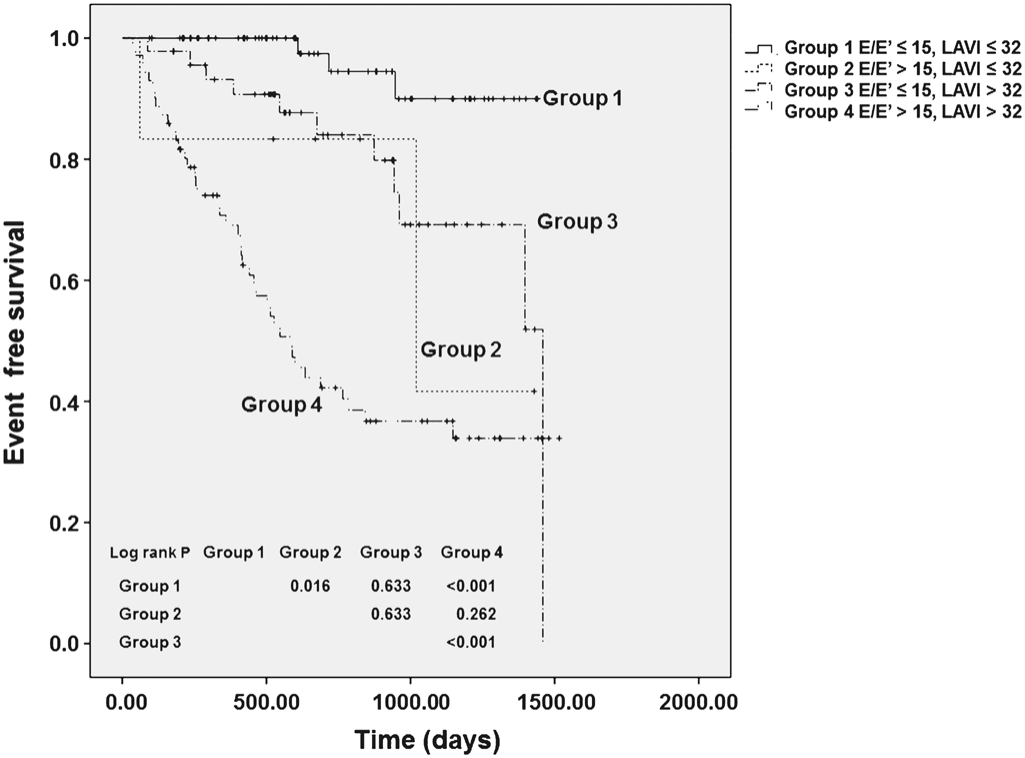
Kaplan-Meier survival curves for CV events according to E/E’ (>15) and LAVI (>32 mL/m^2^).

Patients were classified into 4 groups based on E/E’ and LAVI values. Comparisons were made between the groups based on baseline clinical characteristics and laboratory findings (Table 5). Patients in group 4, defined as E/E’>15 and LAVI>32 mL/m^2^, were significantly older, had significantly higher BMI, systolic BP, prevalence of CAD and arrhythmia, and NT-proBNP concentrations, but a significantly lower 24-hour urine volume compared to those in group 1 (E/E’≤15 and LAVI≤32 mL/m^2^). Moreover, LVEDD, LVMI, E/E’, E/A, LAD, and LAVI were significantly higher, whereas LVEF, E’, and DT were significantly lower in group 4 than in group 1. Furthermore, patients in group 4 had a significantly worse CV event-free survival rate compared to those in groups 1 and 3 (E/E’≤15 and LAVI>32 mL/m^2^), and the CV event-free survival rate was worst in group 4 (log-rank test, *P*<. 001) (Fig. 1).

**Table 5:** Baseline characteristics and echocardiographic parameters of the study patients stratified by E/E’ and LAVI.

Variables	Group 1, (n = 71) E/E’ ≤15, LAVI ≤32	Group 2, (n = 6) E/E’ >15, LAVI ≤32	Group 3, (n = 46) E/E’ ≤15, LAVI >32	Group 4, (n = 71) E/E’ >15, LAVI >32	*P*
Age (years)	52.44 ± 15.95	64.50 ± 13.59	59.52 ± 13.39	61.73 ± 12.20	0.001
Sex (male)	46 (64.8)	0 (0)	30 (65.2)	38 (53.5)	0.011
Dialysis modality (HD)	51 (71.8)	5 (83.3)	39 (84.8)	60 (84.5)	0.208
BMI (kg/m2)	22.88 ± 3.23	22.49 ± 2.87	23.54 ± 3.88	24.45 ± 3.18	0.041
Smoking (Yes)	9 (12.7)	0 (0)	10 (21.7)	10 (14.1)	0.384
Systolic BP (mmHg)	137.65 ± 17.70	136.67 ± 15.25	143.60 ± 18.87	144.72 ± 19.37	0.009
Diastolic BP (mmHg)	79.53 ± 12.31	82.83 ± 12.92	75.50 ± 12.10	76.59 ± 14.07	0.244
Primary cause of renal disease					0.722
Diabetes	27 (38.0)	4 (66.7)	24 (52.2)	36 (50.7)	
Hypertension	16 (22.5)	2 (33.3)	12 (26.1)	14 (19.7)	
GN	10 (14.1)	0 (0)	4 (8.7)	9 (12.7)	
Others	4 (5.6)	0 (0)	2 (4.3)	4 (5.6)	
Unknown	14 (19.7)	0 (0)	4 (8.7)	8 (11.3)	
Comorbid disease					
Chronic lung disease	5 (7.0)	1 (16.7)	2 (4.3)	12 (17.1)	0.096
Coronary arterial disease	6 (8.5)	0 (0)	4 (8.7)	17 (23.9)	0.021
Arrhythmia	0 (0)	1 (16.7)	2 (4.3)	7 (9.9)	0.033
Peripheral arterial disease	11 (15.5)	1 (16.7)	7 (15.2)	14 (19.7)	0.899
Cerebrovascular disease	8 (11.3)	2 (33.3)	7 (15.2)	18 (25.4)	0.113
Ulcer	6 (8.5)	1 (9.1)	0 (0)	4 (5.6)	0.161
Liver disease	4 (5.6)	0 (0)	2 (4.3)	3 (4.2)	0.641
DM	34 (47.9)	4 (66.7)	27 (58.7)	46 (64.8)	0.217
Modified CCI	4.74 ± 2.85	5.67 ± 1.75	5.40 ± 2.36	5.56 ± 2.61	0.133
Davies score	0.90 ± 0.85	1.40 ± 0.89	1.02 ± 0.78	1.24 ± 1.12	0.055
Baseline laboratory findings					
Hemoglobin (g/dL)	9.00 ± 1.17	9.48 ± 0.85	8.51 ± 0.92	9.70 ± 9.64	0.083
Blood urea nitrogen (mg/dL)	61.26 ± 31.81	44.53 ± 30.38	58.80 ± 25.02	54.15 ± 26.93	0.223
Serum creatinine (mg/dL)	7.86 ± 2.64	5.71 ± 2.67	7.80 ± 3.53	16.12 ± 1.91	0.131
Serum albumin (g/dL)	3.27 ± 0.56	2.88 ± 0.46	3.65 ± 2.77	3.19 ± 0.60	0.102
Total cholesterol (mg/dL)	147.53 ± 38.66	129.66 ± 27.60	152.82 ± 41.34	152.29 ± 43.16	0.343
Triglyceride (mg/dL)	103.04 ± 46.73	85.50 ± 27.47	115.30 ± 53.16	124.22 ± 46.49	0.208
LDL-C (mg/dL)	83.75 ± 26.27	73.50 ± 22.29	82.44 ± 33.19	79.86 ± 30.82	0.757
HDL-C (mg/dL)	40.70 ± 11.77	41.33 ± 21.57	37.78 ± 14.30	35.01 ± 9.66	0.253
Sodium (mmol/L)	138.21 ± 3.88	134.33 ± 3.67	138.50 ± 2.54	136.76 ± 4.18	0.032
Potassium (mmol/L)	4.02 ± 0.69	3.40 ± 0.33	4.15 ± 0.67	4.20 ± 0.89	0.851
Bicarbonate (mmol/L)	23.25 ± 4.96	24.00 ± 2.00	22.76 ± 3.60	22.38 ± 4.31	0.779
iPTH (pmol/L)	188.82 (100.0–230.7)	82.76 (25.01–146.47)	217.40 (104.50–223.30)	196.50 (91.32–272.19)	0.358
hs-CRP (mg/L)	12.98 (1–8.04)	16.78 (6.19–28.75)	11.76 (1.13–16.98)	21.75 (1.10–28.17)	0.203
NT-proBNP (pg/mL)	5692 (618–8482)	16572 (4277–35000)	11283 (2354–21047)	17157 (5223–35000)	<0.001
cTnT (ng/mL)	0.06 (0.020–0.09)	0.072 (0.020–0.151)	0.106 (0.030–0.102)	0.138 (0.047–0.130)	0.142
24hr-urine related parameter					
Urine volume (mL/day)	1078.6 (640.0–1400.0)	698.3 (515.0–837.5)	760.9 (430.0–1025.0)	665.1 (375.0–890.0)	0.005
Urine protein (mg/day)	2265.9 (658.9–2966.0)	2239.2 (1020.4–3053.3)	1622.9 (385.8–2077.7)	2236.1 (559.8–2793.7)	0.424
Echocardiography parameters					
LVEF (%)	65.42 ± 7.05	65.66 ± 8.76	66.20 ± 7.06	62.18 ± 8.22	0.019
LVEDD (mm)	49.67 ± 5.29	48.16 ± 6.30	54.83 ± 5.40	54.76 ± 6.29	<0.001
LVESD (mm)	33.63 ± 5.43	32.17 ± 6.55	36.93 ± 6.30	37.83 ± 5.86	<0.001
LVMI (g/m^2^)	121.84 ± 29.29	125.53 ± 34.54	145.18 ± 35.86	152.03 ± 30.98	<0.001
E/E’	10.59 ± 2.29	21.83 ± 6.36	12.13 ± 2.17	22.68 ± 6.19	<0.001
E’ (cm/sec)	6.00 ± 2.39	4.00 ± 1.79	6.13 ± 1.56	4.58 ± 1.22	<0.001
E/A	0.87 ± 0.34	0.84 ± 0.43	1.04 ± 0.57	1.17 ± 0.68	0.015
DT (msec)	222.80 ± 68.58	237.00 ± 66.22	204.54 ± 55.71	193.98 ± 58.48	0.031
LAD (mm)	36.21 ± 5.50	37.00 ± 3.63	45.17 ± 7.43	46.30 ± 5.66	<0.001
LAVI (mL/m^2^)	26.13 ± 6.01	29.71 ± 2.40	46.60 ± 18.50	51.06 ± 17.75	<0.001
RVSP (mmHg)	25.24 ± 7.70	27.83 ± 10.26	30.78 ± 10.75	35.55 ± 13.74	<0.001
RAP (mmHg)	5.38 ± 2.07	6.67 ± 2.58	6.70 ± 4.27	6.48 ± 2.85	0.070

*HD*, hemodialysis; *BMI*, body mass index; *BP*, blood pressure; *GN*, glomerulonephritis; *DM*, diabetes mellitus; *CCI*, Charlson comorbidity index; *HDL-C*, high-density lipoprotein cholesterol; *LDL-C*, low-density lipoprotein cholesterol; *iPTH*, intact parathyroid hormone; *hs-CRP*, high-sensitivity C reactive protein; *NT-proBNP*, N-terminal pro B-type natriuretic peptide; *cTnT*, cardiac troponin T; *LVEF*, left ventricular ejection fraction; *LVEDD*, left ventricular end diastolic diameter; *LVESD*, left ventricular end systolic diameter; *LAD*, left atrial diameter; *LAVI*, left atrial volume index; *LVMI*, left ventricular mass index; *DT*, deceleration time; *RVSP*, right ventricular systolic pressure; *RAP*, right atrial pressure

Data are expressed as mean ± SD, number (percentage), or median (range).

### Clinical, laboratory, and echocardiographic findings as independent risk factors for CV events

**Fig 2:**
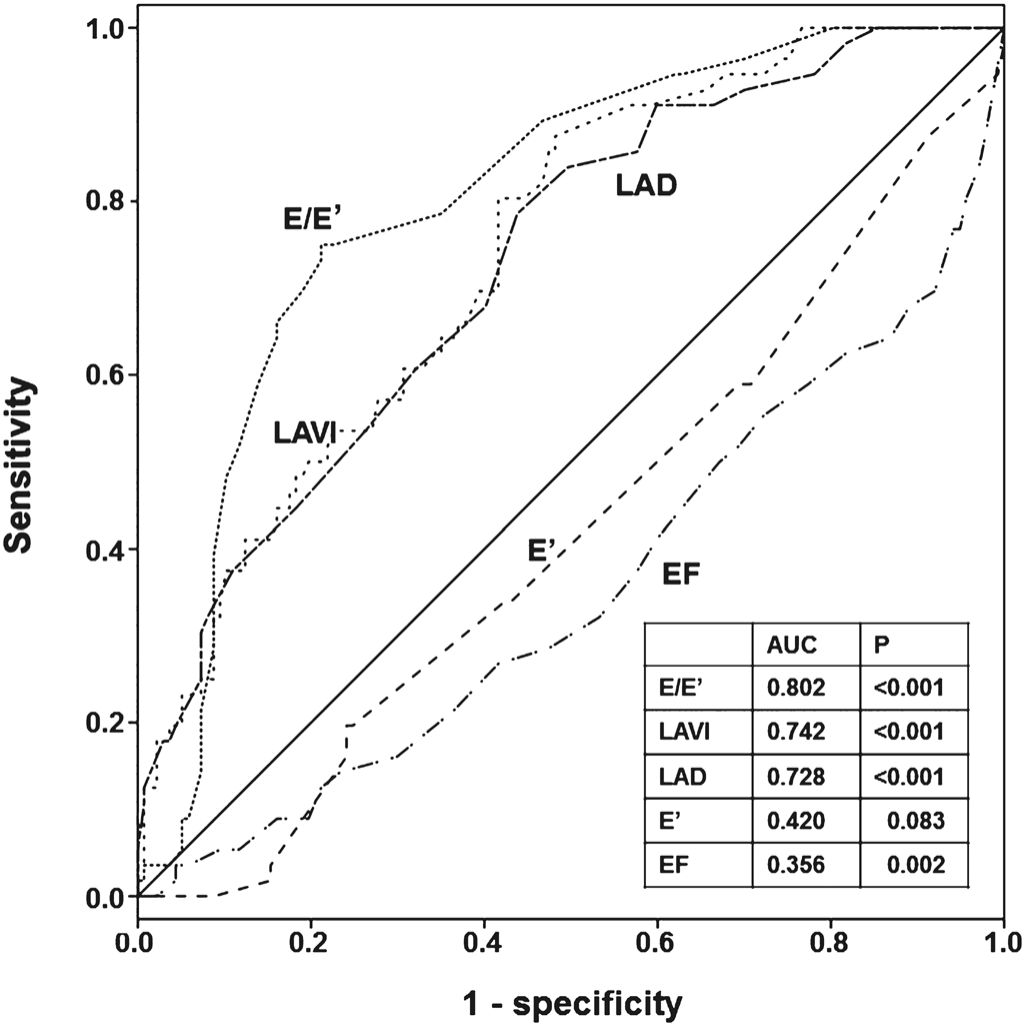
Receiver operating characteristic curve analysis of E/E’, LAVI, LAD, E’, and EF for CV event.

Univariate Cox proportional hazards analysis revealed that clinical and laboratory findings such as age, history of DM, CAD, cerebrovascular disease, arrhythmia, serum albumin and NT-proBNP levels, and 24-hour urine volume were significantly associated with CV events. Among the echocardiographic parameters, LVEF, E/E’, E’, LAD, and LAVI were significant risk factors for CV events. In addition, E/E’>15, LAD>45 mm, and LAVI>32 mL/m^2^ had significant associations with CV events (Table 6). In multivariate Cox analysis, LVEF, E/E’, LAVI, E/E’>15, and LAVI>32 mL/m^2^ were shown to be significant independent predictors of CV events even after adjusting for age, sex, dialysis modality, smoking, history of DM, CAD, CVD and arrhythmia, hemoglobin and serum albumin concentrations, and 24-hour urine volume. Among these parameters, E/E’>15 and LAVI>32 mL/m^2^ had significant power to predict CV events (E/E’>15: hazard ratio [HR] = 5.40, 95% confidence interval [CI] = 2.73–10.70, *P* <. 001; LAVI>32 mL/m^2^: HR = 5.56, 95% CI = 2.28–13.59, *P* <. 001) (Table 7). In addition, E/E’ and LAVI provided higher predictive values for CV events than other echocardiographic parameters such as LVEF and E’ (E/E’: AUC = 0.802, *P* <. 001; LAVI: AUC = 0.742, *P* <. 001) (Fig. 2). To determine whether the impact of E/E’ and LAVI on the clinical outcome was differential according to dialysis modality or the presence of DM, additional Cox proportional analyses were performed. When the analysis was conducted in HD and PD patients separately, either E/E’>15 or LAVI>32 mL/m^2^ was revealed to be a significant independent risk factor for CV events, even after adjusting for confounding factors. Coexistence of E/E’>15 and LAVI>32 mL/m^2^ also had a significant power to predict CV events (HR = 5.34, 95% CI = 2.67 to 10.65, *P* < 0.001) in HD patients, which were consistent with the results of all the patients. However, in PD patients, E/E’>15 and/or LAVI>32 mL/m^2^ were not significant predictors for CV events. We surmised that it was due to a small number of patients (n = 39) and CV events (n = 7) in PD patients. Further multivariate Cox analysis showed that E/E’>15, LAVI>32 mL/m^2^, and coexistence of E/E’>15 and LAVI>32 mL/m^2^ were significant independent predictors of CV events in both DM and non-DM groups, even after adjusting for age, sex, dialysis modality, smoking, history of CAD, CVD and arrhythmia, hemoglobin and serum albumin concentrations, and 24-hour urine volume (Table 8).

**Table 6:** Univariate Cox proportional hazards regression analysis for CV events.

Variables	HR (95% CI)	*P*
Age (years)	1.026 (1.007–1.046)	0.008
Sex (male)	0.989 (0.583–1.676)	0.966
Dialysis modality (HD)	1.983 (0.890–4.418)	0.094
Smoking (Yes)	0.871 (0.426–1.781)	0.705
DM	1.771 (1.001–3.131)	0.049
Coronary arterial disease	2.951 (1.599–5.446)	0.001
Arrhythmia	3.524 (1.591–7.802)	0.002
Cerebrovascular disease	2.782 (1.598–4.844)	<0.001
Hemoglobin (g/dL)	1.015 (0.992–1.038)	0.205
Serum albumin (g/dL)	0.525 (0.334–0.825)	0.005
NT-proBNP (pg/mL)	1.000 (1.000–1.001)	0.002
cTnT (ng/mL)	2.174 (0.861–5.489)	0.100
24-hr urine volume (mL/day)	0.999 (0.998–1.000)	0.001
LVEF (%)	0.948 (0.917–0.981)	0.002
LVEDD (mm)	1.020 (0.985–1.057)	0.263
LVESD (mm)	1.013 (0.910–1.127)	0.813
LVMI (g/m^2^)	1.006 (0.999–1.013)	0.104
E/E’	1.068 (1.040–1.097)	<0.001
E’ (cm/sec)	0.839 (0.709–0.993)	0.041
E/A	0.899 (0.654–1.431)	0.654
DT (msec)	0.996 (0.991–1.000)	0.059
LAD (mm)	1.860 (1.466–2.360)	<0.001
LAVI (mL/m^2^)	1.019 (1.011–1.028)	<0.001
E/E’ >15	5.954 (3.253–10.900)	<0.001
LAD >45 (mm)	2.286 (1.351–3.867)	0.002
LAVI >32 (mL/m^2^)	6.243 (2.676–14.563)	<0.001

*CV*, cardiovascular; *HD*, hemodialysis; *DM*, diabetes mellitus; *NT-proBNP*, N-terminal pro B-type natriuretic peptide; *cTnT*, cardiac troponin T; *LVEF*, left ventricular ejection fraction; *LVEDD*, left ventricular end diastolic diameter; *LVESD*, left ventricular end systolic diameter; *LVMI*, left ventricular mass index; *LAD*, left atrial diameter; *LAVI*, left atrial volume index; *DT*, deceleration time

**Table 7:** Multivariate Cox proportional hazards regression analysis for CV events.

	Model 1		Model 2		Model 3		Model 4		Model 5		Model 6	
	HR 95% CI	*P*	HR 95% CI	*P*	HR 95% CI	*P*	HR 95% CI	*P*	HR 95% CI	*P*	HR 95% CI	*P*
Echocardiographic data												
LVEF	0.95 (0.92–0.99)	0.007										
E/E’			1.05 (1.02–1.09)	0.003								
LAVI					1.02 (1.00–1.03)	0.012						
E/E’ > 15							5.40 (2.73–10.70)	<0.001				
LAVI > 32									5.56 (2.28–13.59)	<0.001		
E/E’>15, LAVI>32											5.96 (3.09–11.51)	<0.001
Clinical and laboratory data												
Age	1.00 (0.98–1.03)	0.843	0.99 (0.97–1.02)	0.998	0.99 (0.98–1.02)	0.909	0.99 (0.97–1.02)	0.657	0.99 (0.97–1.02)	0.742	0.99 (0.97–1.02)	0.997
Sex(male)	0.99 (0.56–1.77)	0.997	1.16(0.63–2.11)	0.636	1.07 (0.59–1.94)	0.836	1.48 (0.79–2.77)	0.218	1.00 (0.56–1.80)	0.998	1.27 (0.69–2.33)	0.437
Dialysis modality (HD)	1.44 (0.61–3.39)	0.404	1.36(0.59–3.15)	0.474	1.36 (0.59–3.15)	0.474	1.69(0.73–3.91)	0.225	1.56 (0.67–3.66)	0.306	1.58 (0.68–3.68)	0.285
Smoking (Yes)	0.87 (0.40–1.91)	0.730	0.94 (0.42–2.11)	0.873	0.71 (0.32–1.58)	0.395	1.18 (0.51–2.77)	0.701	0.69 (0.31–1.51)	0.353	1.05 (0.45–2.44)	0.914
DM	0.96 (0.51–1.98)	0.911	0.95(0.50–1.80)	0.880	1.06 (0.57–1.96)	0.860	0.93 (0.49–1.80)	0.838	0.85(0.45–1.61)	0.613	0.82 (0.42–1.58)	0.543
CAD	1.71 (0.79–3.70)	0.170	1.76 (0.81–3.80)	0.154	2.26 (1.08–4.71)	0.03	1.09 (0.49–2.35)	0.825	1.70 (0.80–3.63)	0.169	1.05 (0.49–2.24)	0.913
CVD	2.23 (1.10–4.45)	0.027	1.99 (0.97–4.08)	0.062	1.94 (0.96–3.89)	0.063	2.45 (1.15–5.22)	0.021	2.60 (1.26–5.39)	0.010	2.77 (1.34–5.73)	0.006
Arrhythmia	2.77 (1.10–7.00)	0.031	1.57 (0.61–4.04)	0.351	1.04 (0.34–3.16)	0.396	2.17 (0.86–5.45)	0.1	1.47 (0.59–3.69)	0.407	2.00 (0.79–5.05)	0.142
Hb	1.02 (0.99–1.05)	0.097	0.99 (0.97–1.03)	0.955	1.01 (0.99–1.04)	0.396	1.00 (0.98–1.03)	0.881	1.01 (0.98–1.03)	0.541	0.93 (0.98–1.03)	0.928
Albumin	0.51 (0.30–0.90)	0.017	0.58 (0.34–0.99)	0.047	0.43 (0.25–0.74)	0.002	0.68 (0.40–1.16)	0.153	0.41 (0.23–0.72)	0.002	0.52 (0.30–0.90)	0.019
24-hr Urine volume	0.99 (0.99–1.00)	0.003	0.99 (0.99–0.99)	0.001	0.99 (0.99–1.00)	0.009	0.99 (0.99–1.00)	0.001	0.99 (0.99–1.00)	0.009	0.99 (0.99–1.00)	<0.001

*CV*, cardiovascular; *HR*, hazard ratio; *CI*, confidential interval; *LVEF*, left ventricular ejection fraction; *LAVI*, left atrial volume index; *DM*, diabetes mellitus; *CAD*, coronary arterial disease; *CVD*, cerebrovascular disease; *Hb*, hemoglobin.

**Table 8:** Multivariate Cox proportional hazards regression analysis for CV events according to dialysis modality or the presence of DM.

	Crude		Model 1	
	HR 95% CI	*P*	HR 95% CI	*P*
**Dialysis modality**				
**HD**				
E/E’>15	6.13 (3.20–11.75)	<0.001	5.56 (2.64–11.70)	<0.001
LAVI>32	6.64 (2.63–16.73)	<0.001	5.97 (2.24–15.86)	<0.001
E/E’>15, LAVI>32	5.50 (2.96–10.22)	<0.001	5.34 (2.67–10.65)	<0.001
**PD**				
E/E’>15	4.92 (0.95–25.65)	0.058	2.96 (0.17–50.87)	0.454
LAVI>32	4.15 (0.48–35.80)	0.195	2.31 (0.05–111.24)	0.671
E/E’>15, LAVI>32	5.26 (1.01–27.40)	0.049	1.01 (0.11–8.76)	0.999
**The presence of DM**				
**DM**				
E/E’>15	5.20 (2.47–10.95)	<0.001	4.05 (1.74–9.41)	0.001
LAVI>32	10.53 (2.54–43.68)	0.001	10.10 (2.23–45.68)	0.003
E/E’>15, LAVI>32	5.90 (2.81–12.42)	<0.001	5.61 (2.46–12.77)	<0.001
**Non-DM**				
E/E’>15	6.75 (2.37–19.22)	<0.001	6.74 (2.07–21.92)	0.002
LAVI>32	3.39 (1.10–10.41)	0.033	3.71 (1.03–14.50)	0.042
E/E’>15, LAVI>32	4.17 (1.58–10.99)	0.004	4.29 (1.45–12.69)	0.008

*CV*, cardiovascular; *HR*, hazard ratio; *CI*, confidential interval; *HD*, hemodialysis; *LAVI*, left atrial volume index; *PD*, peritoneal dialysis; *DM*, diabetes mellitus.

Model 1: adjusted for age, sex, dialysis modality, smoking, history of DM, CAD, CVD and arrhythmia, hemoglobin and serum albumin concentrations, and 24-hour urine volume.

## Discussion

Previous studies have found that diastolic dysfunction, defined as increased E/E’ and/or high LAVI, is an independent predictor of CV morbidity and mortality in the general population and, more specifically, in ESRD patients on chronic dialysis.[6,13,14,17,18,20] However, the impact of diastolic dysfunction on the clinical outcomes has never been investigated in incident dialysis patients with preserved systolic function. The results of the current study show that E/E’>15 and LAVI>32 mL/m^2^ are independent predictors of CV events in this population. In addition, patients with both E/E’>15 and LAVI>32 mL/m^2^ have the worst CV outcomes compared to those with either E/E’>15 or LAVI>32 mL/m^2^.

Diastolic dysfunction is frequently observed in ESRD patients on dialysis. It is also well known that diastolic dysfunction is closely associated LVH.[21] LVH is known to occur in >70% of incident ESRD patients and increases the risk for cardiovascular event in patients on dialysis.[22–25] Moreover, LVH has been revealed to be a significant independent predictor of CV outcome in patients with ESRD. Furthermore, the change in LVH has been found to be a strong prognostic factor in these patients.[26] A previous prospective study on 161 prevalent HD patients showed that the rates of the increase in LVMI were significantly higher in patients with incident CV events compared to those without such events; moreover, the relative risk of adverse CV outcomes was significantly higher in patients with changes in LVMI above the 75^th^ percentile than in those with changes below the 25^th^ percentile (HR = 2.01, 95% CI = 1.46–2.54).[27] Similarly, in a cohort study of 153 incident HD patients, the HRs associated with a 10% reduction in LV mass were 0.78 for all-cause mortality and 0.72 for CV mortality during a mean follow-up duration of 54 months.[28] In that study, a partial regression of LV mass also resulted in improved patient survival even after adjustment for age, gender, DM, history of CV disease, baseline LVMI, and all nonspecific CV risk factors. However, the results of the present study failed to show an independent prognostic effect of LVMI on CV events in incident dialysis patients with preserved systolic function. Most of the aforementioned studies did not include E/E’ and LAVI in their analysis, and LVMI had a significant positive correlation with E/E’ and LAVI in our study. Therefore, the influence of LVMI on clinical outcomes was attributed to diastolic dysfunction rather than LVH per se. In a similar context, we have previously demonstrated that LAVI, unlike LVMI, was an independent risk factor for all-cause and CV mortality in 216 PD patients, when LVMI, E/E’, and LAVI were included in the analysis.[17] Kim et al. also found that E/E’ and LAVI were significant independent predictors of the decline of residual renal function, and E/E’>15 was significantly associated with future CV events in 82 incident PD patients, while LVH was not.[20]

Accumulating evidence indicates that CAD is another risk factor for diastolic dysfunction. Although previous studies have shown that CAD is less common in diastolic HF patients compared to HF patients with reduced EF,[29] the prevalence of CAD is not low. O’Connor et al. have shown that CAD was present in 65% of 2,498 patients with New York Heart Association class II to IV symptoms and EF>40%.[30] In addition, a large cohort study of patients from the Acute Decompensated Heart Failure National Registry (ADHERE) has demonstrated that 50% of 26,322 HF patients with preserved systolic function had CAD.[31] Moreover, Sharma et al. found that the prevalence of severe CAD was 52% in 38 ESRD patients, whose LV end-diastolic pressure was ≥15 mmHg at cardiac catheterization.[13] In the current study, only 23.9% of patients with E/E’>15 and LAVI>32 mL/m^2^ had a history of CAD. Kim et al. also previously showed that CAD was present in 17.4% of the 46 incident PD patients with LAVI>32 mL/m^2^.[32] We infer that the discrepancy in the prevalence of CAD can be attributed to differences in patient age, ethnicity, BMI, and duration of dialysis. Moreover, the proportion of patients with CAD was significantly higher in patients with CV events compared to the CV event-free group (24.6% vs. 9.5%, *P* = .008). Furthermore, univariate Cox analysis revealed that CAD was significantly associated with CV events. However, the significant impact of CAD on CV events disappeared when diastolic echocardiographic parameters were included in the multivariate analysis, whereas the predictive power of E/E’ and LAVI for CV events remained significant even after adjusting for CAD. Based on these findings, it is presumed that diastolic dysfunction is a more significant risk factor for future CV events than a history of CAD and seems to partly contribute to the occurrence of CAD in Korean incident dialysis patients with preserved EF, among whom severe CAD was less prevalent.

The gold standard assessment of diastolic function is measuring the mean pulmonary capillary wedge pressure and LV end-diastolic pressure by cardiac catheterization; the mean pulmonary capillary wedge pressure of >12 mmHg or LV end-diastolic pressure of >16 mmHg is considered high LV filling pressure, indicating diastolic dysfunction.[33] However, it is irrational to routinely perform an invasive procedure in the clinical field only for the evaluation of diastolic function. Therefore, noninvasive Doppler echocardiographic parameters, such as the E/A ratio, E’, E/E’ and LAVI, have been used to estimate the LV filling pressure. Among those, E/E’ and LAVI have been shown to reliably assess diastolic function in dialysis patients, as well as in the general population. In a study of 100 patients with suspected CV disease referred for cardiac catheterization, E/E’ provided a better estimation of the mean LV end-diastolic pressure—a surrogate for mean LA pressure—compared to other Doppler parameters.[34] Furthermore, E/E’ but not CAD, NT-proBNP, and LA size was shown to be an independent predictor of increased LV filling pressure in 125 renal transplant candidates.[13] However, LAVI has been regarded as a marker of the severity and duration of diastolic dysfunction. LAVI was progressively increased as diastolic dysfunction worsened, and was significantly correlated with the severity of diastolic dysfunction. In addition, some investigators have suggested that Doppler parameters only provide information about LV filling at the time of measurement, while increased LAVI often reflects the cumulative effect of filling pressure over time.[18,35,36] Despite the presence of numerous previous studies, there is still a controversy on which parameter, such as E/E’ or LAVI, provides a better predictive value for diastolic dysfunction and which parameter is a more powerful prognostic factor for clinical outcomes. Based on these findings, it has been suggested that integrating multiple echocardiographic indices is reasonable to diagnose and grade diastolic dysfunction. For example, an integration of the E/A, E’, E/E’, and LAVI parameters has been used by the American Society of Echocardiography to classify diastolic dysfunction into three groups: impaired LV relaxation (grade I), pseudonormal LV filling (grade II), and restrictive LV filling (grade III), by using E/A, E’, E/E’, and LAVI.[33] This grading system significantly predicted patients’ mortality in not only the general population but also ESRD patients on chronic hemodialysis. Our study findings are consistent with those from previous studies, showing that incident ESRD patients with both E/E’>15 and LAVI>32 mL/m^2^—corresponding to grade III diastolic dysfunction—had the worst clinical outcomes. Moreover, the predictive value of E/E’ plus LAVI for CV events was higher than either E/E’ or LAVI. Based on these findings, it is suggested that an assessment of diastolic dysfunction using multiple echocardiographic indices can increase the predictive power of future CV events in dialysis patients.

In earlier studies, LVEF provided valuable information on ESRD patients on dialysis.[37,38] Furthermore, it was found to be a significant risk factor for all-cause and CV morbidity and mortality in these patients. In addition, both systolic and diastolic dysfunction by progressive impairment in contractility and stiffening of the myocardial wall, respectively, is known to occur in the late stage of LVH and cardiac fibrosis.[9] Therefore, the independent effect of diastolic dysfunction on clinical outcomes remains to be clarified in patients with preserved LV systolic function. However, most of the previous studies on the association between diastolic dysfunction and patient outcomes did not exclude patients with systolic dysfunction or only excluded patients with severely low LVEF. In contrast, the present study was restricted to patients with preserved LVEF, thus facilitating the investigation of the independent effect of diastolic dysfunction in incident ESRD patients.

Several limitations of this study should be discussed. First, since our study subjects were all Korean incident ESRD patients, the association of diastolic dysfunction with CV events may not be generalizable to other populations. Second, because echocardiographic examinations were performed only at the baseline, it was difficult to determine the consequence of the changes of echocardiographic parameters on patients’ clinical outcomes. In addition, echocardiography alone was used to assess diastolic function, and thus it might be unsatisfactory to precisely define diastolic dysfunction. New methods, such as gated radionuclide left ventriculography and phosphorus-31 magnetic resonance spectroscopy, may be useful to evaluate early stage myocardial metabolic abnormalities.[39,40] Third, the number of patients was not large and the number of mortality was too small to elucidate the impact of E/E’ and/or LAVI on CV mortality in the current study. Fourth, the follow-up duration was relatively short. Since these patients have continuously been followed up, a better long-term study will be performed in the near future. Fifth, treatments known to influence diastolic dysfunction, including blood pressure control, use of diuretics, blockade of the renin-angiotensin system, correction of anemia, and coronary revascularization were not considered in our analysis. Sixth, even though routine chest X-rays and physical examinations were performed to judge the volume status of the study subjects, and echocardiography was done when the physicians considered their patients to be clinically euvolemia in the present study, we did not perform objective body fluid monitoring techniques such as bioimpedance, or inferior vena cava diameter or continuous blood volume measurements, which might provide more convincing results. Moreover, target dry weights of each patient were determined based on physicians’ judgments. Therefore, we could not completely exclude the possibility of hypervolemia and hypovolemia at the time of echocardiography. In the future, repetitive assessment and objective determination of the dry weights of each patient will be needed to ascertain the exact body fluid balance. Finally, although the levels of NT-proBNP—a biomarker recommended mainly for the exclusion of cases of diastolic HF with normal EF—were determined and included in the analysis, we only included EF values in the inclusion criteria in our study.

In conclusion, an increase in E/E’ or LAVI is a significant risk factor for CV events in incident ESRD patients with preserved LV systolic function. Our findings suggest that diastolic dysfunction, assessed by Doppler echocardiography, may be useful in stratifying CV event risk and providing a therapeutic direction for the management of these patients.

## Supporting Information

S1 Fig Flow diagram of patient selection and outcomes. From July 2008 to August 2012, 293 patients who started HD or PD at Yonsei University Health System were initially recruited for enrollment, and 194 patients were included in the final analysis after excluding 24 patients who did not undergo echocardiography and 75 patients who met the exclusion criteria. Patients were divided into two groups according to the presence of CV events; CV event-free group and CV event group.
(TIF)

## Abbreviations

LV: left ventricle
CV: cardiovascular
