# Supplementary figures and images for: Diastolic Dysfunction Is an Independent Predictor of Cardiovascular Events in Incident Dialysis Patients with Preserved Systolic Function
Source: PLoS One. 2015 Mar 4;10(3):e0118694. doi: 10.1371/journal.pone.0118694 (PMC4349827; doi:10.1371/journal.pone.0118694)

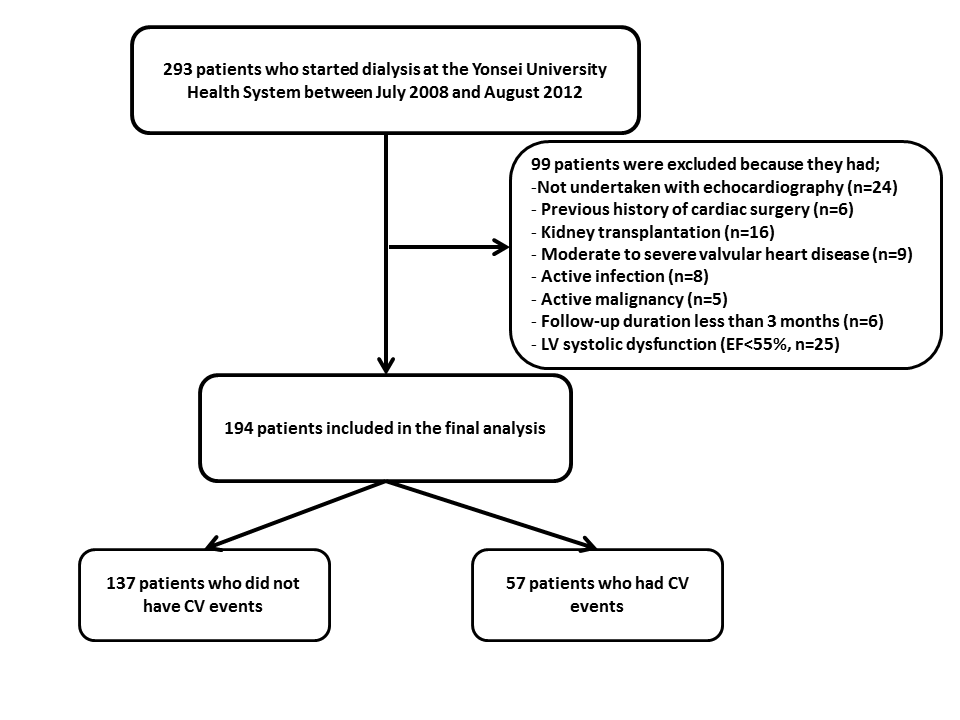

Supplement: S1 Fig — From July 2008 to August 2012, 293 patients who started HD or PD at Yonsei University Health System were initially recruited for enrollment, and 194 patients were included in the final analysis after excluding 24 patients who did not undergo echocardiography and 75 patients who met the exclusion criteria. Patients were divided into two groups according to the presence of CV events; CV event-free group and CV event group. (TIF) [file pone.0118694.s001.tif]
